# Sex Differences in the Metabolome of Alzheimer’s Disease Progression

**DOI:** 10.3389/fradi.2022.782864

**Published:** 2022-03-14

**Authors:** Tomás González Zarzar, Brian Lee, Rory Coughlin, Dokyoon Kim, Li Shen, Molly A. Hall

**Affiliations:** 1 Department of Veterinary and Biomedical Sciences, College of Agricultural Sciences, The Pennsylvania State University, University Park, PA, United States; 2 Huck Institutes of the Life Sciences, The Pennsylvania State University, University Park, PA, United States; 3 Department of Biostatistics, Epidemiology and Informatics, Perelman School of Medicine, University of Pennsylvania, Philadelphia, PA, United States; 4 Penn State Cancer Institute, The Pennsylvania State University, University Park, PA, United States

**Keywords:** Alzheimer’s disease, sex differences, metabolomics, phosphatidylcholine, very low-density lipoprotein (VLDL)

## Abstract

Alzheimer’s disease (AD) is the leading cause of dementia; however, men and women face differential AD prevalence, presentation, and progression risks. Characterizing metabolomic profiles during AD progression is fundamental to understand the metabolic disruptions and the biological pathways involved. However, outstanding questions remain of whether peripheral metabolic changes occur equally in men and women with AD. Here, we evaluated differential effects of metabolomic and brain volume associations between sexes. We used three cohorts from the Alzheimer’s Disease Neuroimaging Initiative (ADNI), evaluated 1,368 participants, two metabolomic platforms with 380 metabolites in total, and six brain segment volumes. Using dimension reduction techniques, we took advantage of the correlation structure of the brain volume phenotypes and the metabolite concentration values to reduce the number of tests while aggregating relevant biological structures. Using WGCNA, we aggregated modules of highly co-expressed metabolites. On the other hand, we used partial least squares regression-discriminant analysis (PLS-DA) to extract components of brain volumes that maximally co-vary with AD diagnosis as phenotypes. We tested for differences in effect sizes between sexes in the association between single metabolite and metabolite modules with the brain volume components. We found five metabolite modules and 125 single metabolites with significant differences between sexes. These results highlight a differential lipid disruption in AD progression between sexes. Men showed a greater negative association of phosphatidylcholines and sphingomyelins and a positive association of VLDL and large LDL with AD progression. In contrast, women showed a positive association of triglycerides in VLDL and small and medium LDL with AD progression. Explicitly identifying sex differences in metabolomics during AD progression can highlight particular metabolic disruptions in each sex. Our research study and strategy can lead to better-tailored studies and better-suited treatments that take sex differences into account.

## 1. INTRODUCTION

Alzheimer’s disease (AD) is a neurodegenerative disease and the most common cause of dementia. In the U.S., 5.7 million people lived with AD in 2018, and it is projected that by 2025, 7.1 million people in the U.S. will have developed AD (1). Most cases of AD and dementia occur in women, particularly in the most elderly (2). For example, in the U.S., out of the 5.5 million people age 65 or older with AD, 3.4 million are women, and only 2 million are men (1). Besides differences in prevalence, other sex differences have been described, particularly in disease risk, presentation, and progression. For example, the principal genetic risk factor of AD, the presence of the *ϵ*4 allele in the apolipoprotein E gene (*APOEϵ*4), confers a greater risk of developing AD in women compared to men (3). Similarly, hippocampus atrophy rates occur faster in women than men (4).

Because metabolic decline is one of the earliest symptoms in AD progression, metabolomics has appeared as a relevant area to identify metabolic disruptions across biofluids (5). Furthermore, technological advances in high-throughput metabolomics instruments have made it easier to measure hundreds of metabolites and gain the ability to take fine-grained snapshots of metabolic profiles during disease progression. Because blood is a non-invasive and readily available biofluid, significant efforts have been made to link changes in cognitive decline with peripheral metabolomic changes in serum or plasma. For example, preclinical biomarker-defined stages of AD have been associated with altered levels of phosphatidylcholines (PCs) and sphingomyelins (SMs), while changes in brain volumes and cognition have been associated with long and short acylcarnitines, valine, and *α* – *AAA* (6). Comparisons between controls, mild cognitive impairment (MCI), and AD participants have shown that polyamine and l-arginine metabolism are implicated in differences across all three diagnostic groups (7). Out of several blood metabolomic studies, the role of lipid homeostasis appears to be fundamental in the development of AD (5).

Some sex differences have been reported previously in the association between metabolites and AD biomarkers. Specifically, acylcarnitines, histidine, valine, and proline have shown greater effect sizes in females, while ether-containing PCs, threonine, asparagine, glycine, and other acylcarnitines have shown greater effects in males (8). These results highlight potential sex-specific roles of energy metabolism and homeostasis in the progression of AD (8).

Notwithstanding these advances, central questions remain. For example, despite that AD progression can be characterized by specific morphological and biomarker changes, its heterogeneity is a hallmark (9). Therefore, in the face of heterogeneous changes, how are sex differences displayed? Here, we analyzed differential associations in metabolites and AD phenotypes between sexes, to highlight metabolite differences in disease progression that contribute to the observed sex differences in AD. Using three cohorts from the Alzheimer’s Disease Neuroimaging Initiative (ADNI), two metabolomics platforms, and brain imaging to summarize AD progression, we evaluate differential associations between sexes in single metabolites, and in metabolite modules that bring together highly correlated metabolites.

## 2. MATERIALS AND METHODS

### 2.1. Study Participants

The data used for this study were obtained from the Alzheimer’s Disease Neuroimaging Initiative (ADNI; adni.loni.usc.edu). ADNI was launched in 2003 as a public-private partnership led by Principal Investigator Michael W. Weiner, MD. The primary purpose of ADNI has been to measure the progression from mild cognitive impairment (MCI) to Alzheimer’s disease (AD) through the use of serial magnetic resonance imaging (MRI), positron emission tomography (PET), other biological markers, and clinical and neuropsychological assessments. For up-to-date information, see www.adni-info.org.

Cohorts ADNI 1, ADNI GO, and ADNI 2 were included for this study, employing imaging and metabolomic data from 1,368 participants. Because participants might be included in more than one ADNI cohort, we only included those measurements taken at baseline; therefore, data from participants included in subsequent cohorts were excluded by definition. For example, for a participant included in the ADNI 1 and ADNI GO cohorts, we only considered the measurement at baseline, in ADNI 1, and not the one in ADNI GO. There were 621 females and 747 males, and 92.5% (*N* = 1,266) self-described as white. Descriptive statistics of basic demographic information, *APOEϵ*4 condition, and diagnosis can be observed in Table 1.

### 2.2. Metabolomics Data Acquisition

Two metabolomics platforms were used in this analysis: the AbsoluteIDQ-p180 metabolomics kit (Biocrates Life Science AG, Innsbruck, Austria) and the NMR metabolomics platform from Nightingale (Nightingale Health Ltd., Helsinki, Finland). The p180 platform is a targeted metabolomics platform that can detect up to 188 metabolites distributed in five different classes. Acylcarnitines, sphingolipids, and glycerophospholipids are analyzed by flow injection analysis-tandem mass spectrometry (FIA-MS/MS), while amino acids and biogenic amines are analyzed using an ultra-performance liquid-chromatography tandem mass spectrometer (UPLC-MS/MS) (10).

The Nightingale platform uses nuclear magnetic resonance (NMR) for untargeted high-throughput detection of diverse metabolites, including routine lipids, lipoprotein subclass profiling with lipid concentrations within 14 subclasses, fatty acid composition, and various low-molecular metabolites, including amino acids, ketone bodies, and gluconeogenesis-related metabolites (11).

### 2.3. Metabolomics Data Processing

The p180 metabolomics data was processed using previously published protocols (6, 8, 10). Thirty-four metabolites were removed for having 20% or more missing values. Cross-plate mean normalization was estimated for each metabolite to correct plate batch effects. Duplicates and triplicates were used to estimate the coefficient of variation (CV) and intra-class correlation (ICC) for each metabolite. Three metabolites with CV greater than 20% and 12 metabolites with ICC less than 65% were removed. Eighty-eight non-fasting participants were removed, and one with missing data greater than 40%. Concentration values in replicates were averaged to obtain a single estimation per metabolite. The missing data source was evaluated, and values were imputed when the missing value was lower than the detection limit. The metabolites taurine and C5:DC:C6:OH were removed because they showed values across several participants greater than the highest calibration standard, and the internal standard was out of range, respectively. All other missing values were due to the concentration being lower than the limit of detection (LOD) or because the concentration value was higher than the LOD but lower than the calibration standard. Therefore, 278 missing data points across 26 metabolites were imputed with half of the LOD value per metabolite per plate. Because some LOD values were zero, a constant value of 1 was added to all metabolite concentration values. Metabolite concentration values were transformed using log2-transformation, z-score normalization, and winsorizing values greater than 3 and −3. Finally, 114 participants were removed based on a multivariate outlier detection using the Mahalanobis distance and a Chi-square of *P* < 0.001.

On the other hand, data processing for the NMR platform involved the removal of five metabolites with 20% or more of missing values, one participant with a missing value greater than 40%, and 80 non-fasting participants. Concentration values in replicates were averaged to obtain a single estimation per metabolite. Various QC tags identified potential sources of contamination of the blood samples. For example, “low ethanol” indicated potential disinfectant contamination. Therefore, 33 participants with missing data with any QC tag except for “below limit of quantification” were removed. The remainder of the missing values were all due to the concentration value being below the limit of quantification; therefore, data were imputed using half of the minimum observed value. A total of 155 data points were imputed across 11 metabolites. Finally, data transformation in concentration values involved the addition of a constant of 1, log2-transformation, and z-score normalization.

Residuals from linear regressions were used to account for medication intake. For each metabolite, a linear regression with the medications as predictors was fitted after using a backward selection strategy to keep only significant ones in the model (10). After data processing, 1,475 participants and 135 metabolites were retained in the p180 platform, and 1,562 participants and 245 metabolites in the NMR platform.

### 2.4. Phenotype and Covariate Data Acquisition and Processing

Volumetric brain data from ventricles, hippocampus, entorhinal cortex, fusiform gyrus, middle temporal gyrus, and the whole brain were obtained from the data prepared for the Alzheimer’s Disease Modeling Challenge in the Quantitative Templates for the Progression of Alzheimer’s disease (QT-PAD). These regions were selected because their volumes have been shown to be affected by AD. Specifically, while all segments, including the whole brain, show atrophy with AD development, ventricles show an enlargement (12). We chose the number and type of brain regions to strike a balance between more insight and interpretability. Volumetric segmentation was performed using the FreeSurfer software (13). ADNI 1 1.5T data was run with FreeSurfer version 4.3, while ADNI 1 3T data was run with FreeSurfer version 5.1. ADNI GO and ADNI 2 cohorts were run with FreeSurfer version 5.1. Finally, to control for differences in intracranial volume (ICV), each brain volume was divided by ICV. The ventricle by ICV volume followed a non-normal distribution and was thus log-transformed.

Covariate information including age, sex, years of education, *APOEϵ*4, and diagnosis were extracted. The six diagnosis categories (control, subjective memory complaints, mild cognitive impairment, early mild cognitive impairment, late mild cognitive impairment, and Alzheimer’s disease) were consolidated into three categories by merging control and subjective memory complaints into controls, and mild cognitive impairment, early mild cognitive impairment, and late mild cognitive impairment into mild cognitive impairment. Participants with missing values in any phenotypes or covariates were removed, resulting in 1,368 participants.

### 2.5. Dimension Reduction Techniques

Dimension reduction approaches were applied to reduce the number of comparisons in the imaging phenotypes and the metabolites. A partial least squares regression-discriminant analysis (PLS-DA) was used to reduce the number of phenotypes while maximizing their covariance with the diagnosis groups, and therefore, with AD progression (14). PLS-DA aims to predict the outcome from a set of predictors by extracting a set of orthogonal components that have the best predictive power (15). The six brain phenotypes were used as the predictors and the three diagnosis categories as outcomes after conversion to a dummy categorical matrix. The first five components that explained 95% of the variance were extracted and used for further analysis. Finally, nine participants were removed based on a multivariate outlier detection using the Mahalanobis distance and a Chi-square of *P* < 0.001.

On the other hand, a weighted correlation network analysis (WGCNA) extracted metabolite modules of highly co-expressed metabolites. Although WGCNA has been generally applied to gene expression data, application to metabolomics platforms has been successful (16-18). WGCNA was applied to both metabolomics platforms independently. First, a similarity network was constructed based on the absolute value of the correlation between metabolites. Then, a soft-threshold power was selected based on the criteria of approximating a scale-free topology to generate the adjacency matrix from the similarity matrix. A soft-thresholding power *β* of 10 was used in both platforms (Supplementary Figure S1). Finally, a topological overlap matrix (TOM) was generated from the adjacency matrix (19).

Hierarchical clustering with a dynamic tree cut approach was used to generate metabolite modules, using a minimum cluster size of 5 for the p180 platform and 10 for the NMR (20). Highly correlated metabolite modules were merged (Pearson’s *r* > 0.9), and the final set of metabolite modules was obtained. Concentration values for each metabolite module were summarized by using the module eigen-metabolite, which is the first principal component from the concentration values (19). Finally, each metabolite’s module membership (MM) value was obtained as the Pearson correlation between the single metabolite concentration value and the module eigen-metabolite.

### 2.6. Association Analysis and Sex Differences Detection

A sex-stratified linear regression was fitted for each metabolite module and every individual metabolite, using the brain volume PLS-DA components as predictors. Age, years of education, and *APOEϵ*4 were used as covariates. Two approaches were used to detect sex differences in the metabolite-brain associations (8, 21).

Winkler et al. (21) have suggested detecting sex differences using the sex difference test in the whole set of associations and in a subset that passes a filtering criterion. The sex difference test is defined by:

$$Z_{diff}=\frac{\beta_{f}-\beta_{m}}{\sqrt{se_{f}^{2}+se_{m}^{2}}}$$

Where *β_f_* is the *β* from a regression in the female cohort, *se_f_* is the standard error, and *Z_diff_* follows a normal distribution. Then, a Bonferroni-corrected *α* is used to detect significant sex differences.

On the other hand, the filtering criterion is defined by a significant metabolite-brain association in the merged cohorts. A significant overall association from two cohorts can be obtained by:

$$Z_{overall}=\frac{\frac{\beta_{f}}{se_{f}^{2}}+\frac{\beta_{m}}{se_{m}^{2}}}{\sqrt{\frac{1}{se_{f}^{2}}+\frac{1}{se_{m}^{2}}}}$$

An *α* = 10^−5^ was used to select significant overall associations. Then, the sex difference test is applied to only this subset of associations using a Bonferroni-corrected *α*. The logic behind this two-fold approach is to increase the power to identify different types of sex differences (21).

On the other hand, Arnold et al. (8) have suggested determining sex differences by selecting the associations that fulfill any of the following criteria: (a) associations Bonferroni significant in the entire cohort; (b) Bonferroni significant associations in one sex; and (c) associations showing nominal significance in one sex (*P* < 0.05) and a significant sex difference test. This subset of associations is then categorized as homogeneous if the sex difference *P* > 0.05 and heterogeneous if the sex difference *P* < 0.05. Associations that are Bonferroni significant in a single sex and with a sex difference *P* < 0.05 are classified as sex-specific.

To adjust for multiple testing in the metabolite module and brain component associations, a Bonferroni corrected *α* = 0.05/(*M* × *C*) was used, where *M* is the number of modules, and *C* is the number of brain phenotype components. The effective number of independent tests was estimated to take into account the correlation structure of single metabolites (22). Therefore, in the single metabolite and brain component associations, a Bonferroni corrected *α* = 0.05/*I* × *C* was used, where *I* is the effective number of independent tests.

### 2.7. Software, Packages, and Code Availability

The analysis was written in python (Python Software Foundation, https://www.python.org/) and R (23), including several packages (24-27). The code is stored in Zenodo, under the doi: 10.5281/zenodo.6049171. A README file contains instructions for replicating the analysis, and a conda environment file indicates the specific packages and versions used.

## 3. RESULTS

### 3.1. Phenotype and Metabolite Dimension Reduction

**FIGURE 1 ∣:**
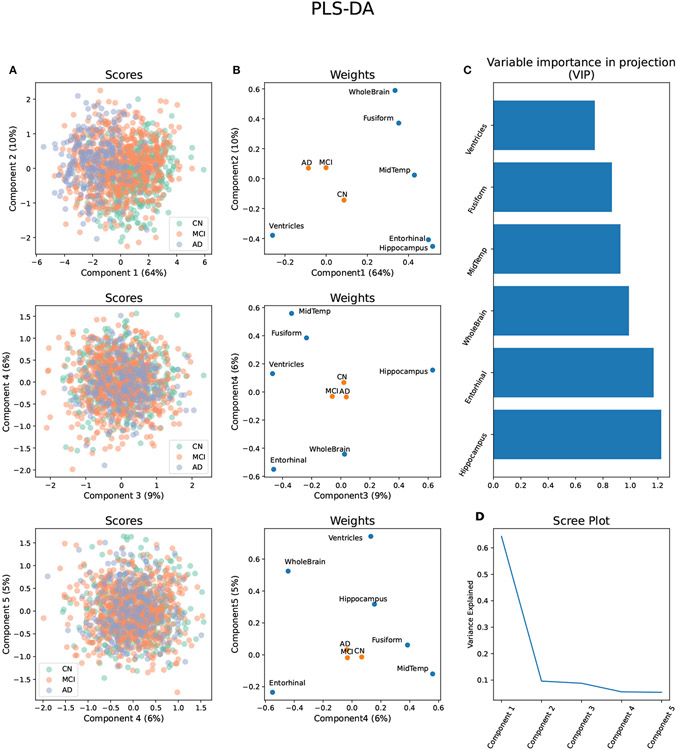
Partial Least Square - Discriminant Analysis (PLS-DA) using six brain volume segments as predictors and diagnosis groups AD, MCI, and CN as outcomes. **(A)** Scores projected in the brain components 1–5 with diagnosis groups color-coded. **(B)** Weights of the predictors and outcomes projected on the brain components 1–5. **(C)** Variable importance in projection (VIP) of each brain volume segment considering the brain components 1–5. **(D)** Scree plot showing the proportion of variance explained by each brain component.

The first five components of the PLS-DA applied to the volumetric brain data distinguished between the three diagnosis groups and explained 95% of the variance (Figures 1A-D). The variable importance in projection (VIP) estimation established that, considering all components, the hippocampus, entorhinal cortex, and whole-brain volumes were the top three segments contributing to the PLS-DA transformation (Figure 1C). All brain segments contributed to brain component 1, which explained 64% of the total brain volume variance and separated all three diagnosis groups . AD progression in brain component 1 was characterized by relative atrophy of most brain segments and an expansion of the ventricles (Figure 1B). On the other hand, the segments that mainly contributed to brain component 2 were the whole-brain volume, followed by the hippocampus and entorhinal cortex volumes. Brain component 2 explained only 10% of the variance in brain volume and only separated AD and MCI from CN. AD progression in brain component 2 was characterized by relative atrophy of the hippocampus, entorhinal cortex , and ventricles and an expansion of the whole brain (Figure 1B).

Brain component 3 explained 9% of the total variance, separated MCI from AD and CN, and its MCI progression was characterized by relative atrophy of the hippocampus and an expansion of the ventricles and the entorhinal cortex. Brain component 4 explained 6% of the total variance in brain volume, separated MCI and AD from CN, and it was characterized by a relative expansion of the entorhinal cortex and the whole brain, and atrophy of the middle temporal gyrus, and the fusiform gyrus. Finally, brain component 5 explained 5% of the variance, separated AD from MCI and CN, and it was characterized by a relative expansion of the ventricles, whole brain, and hippocampus, and atrophy of the entorhinal cortex (Figure 1B).

**FIGURE 2 ∣:**
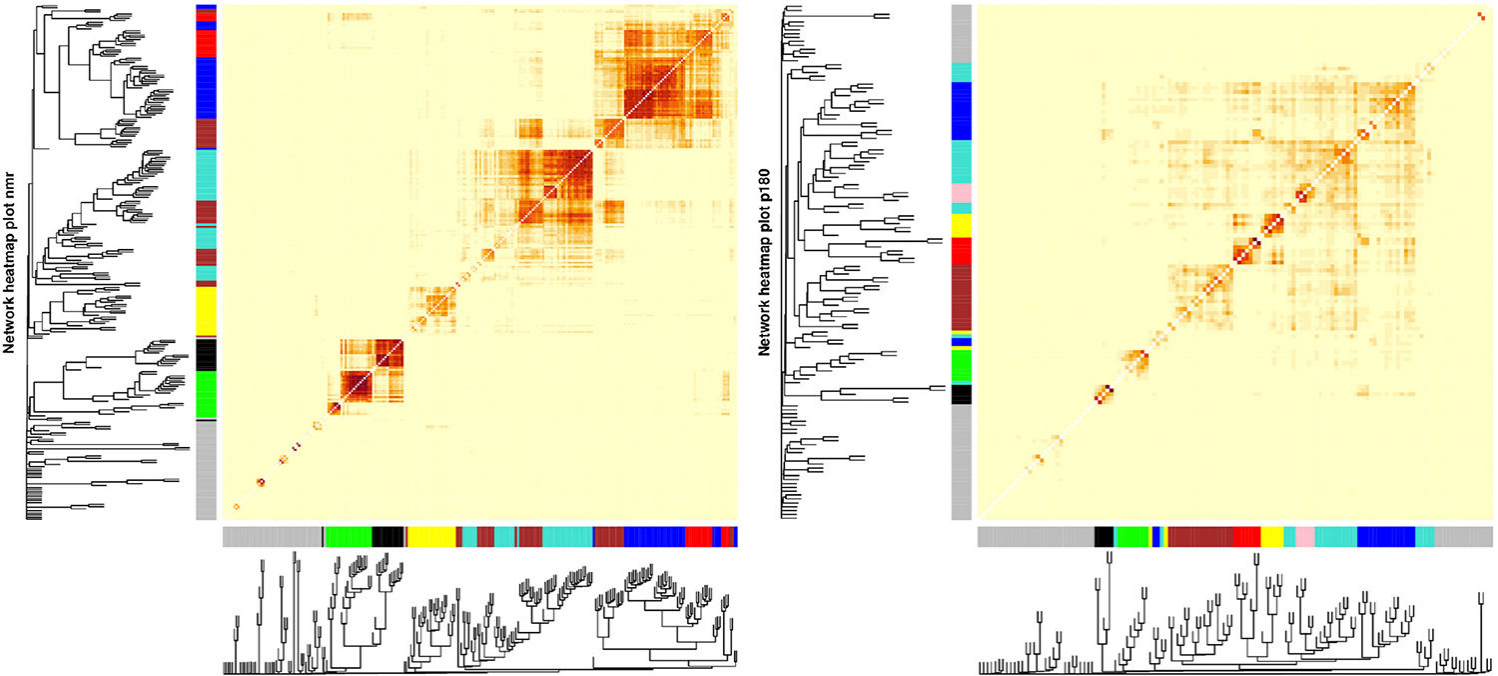
Weighted correlation network analysis (WGCNA) showing the dendrogram, metabolite modules generated indicated by their respective colors, and the topological overlap matrix (TOM) displayed as a heatmap. Red colors in the heatmap indicate greater similarity between the metabolites.

WGCNA applied to the metabolomic platforms produced eight) modules in the p180 platform and seven modules in the NMR platform . In both platforms the gray module contained unassigned metabolites (Figure 2). The average number of metabolites per module in the p180 platform was 11 and 45 metabolites were not assigned and included in the gray module. The largest module was the turquoise with 21 metabolites, and the smallest was the black and pink modules, both with five metabolites. In the p180 platform, the pink, red, turquoise, yellow, black, and blue modules contained mainly various phosphatidylcholines (PC). The brown module contained sphingomyelins, and the green module lysophosphatidylcholines (lysoPC). Finally, the gray module, containing all unassigned metabolites, included amino acids, biogenic amines, and acylcarnitines.

The average number of metabolites per module in the NMR platform was 28. 49 metabolites were unassigned and, therefore, included in the gray module. The largest module was the turquoise with 42 metabolites, and the smallest was the black with sixteen metabolites. The black module mainly contained large and very large high-density lipoproteins (HDL), and the green module included small and medium HDL, particularly cholesterol. On the other hand, the brown module contained all types of very low-density lipoproteins (VLDL), fatty acids, and apolipoproteins. In contrast, the turquoise module included small and medium HDL, specifically various lipid ratios, and large, very large, chylomicrons and extremely large VLDL, and the yellow module contained very small, small, and medium VLDL lipid ratios. Finally, the blue module included primarily small, medium, and large low-density lipoproteins (LDL), fatty acids, and apolipoproteins, and the red module contained mainly intermediate-density lipoprotein (IDL) and other lipids such as PC and sphingomyelins. The unassigned metabolites were mostly ketone bodies, amino acids, glycolysis-related metabolites, and fatty acids.

### 3.2. Detection of Sex Differences

None of the associations, either using single metabolites or metabolite modules in the p180 and NMR platforms, were identified as different between sexes using the approach from Winkler et al. (21). On the other hand, using the Arnold et al. (8) approach resulted in various associations categorized as heterogeneous between sexes and sex-specific.

#### 3.2.1. P180 Platform

**FIGURE 3 ∣:**
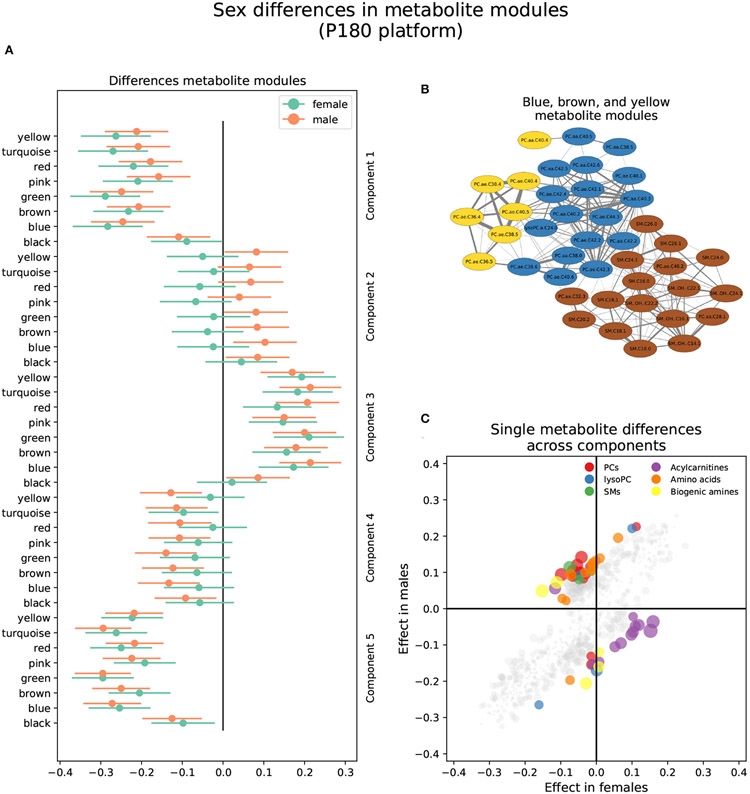
Sex differences in the p180 metabolomics platform. **(A)** Effect sizes and their 95% confidence interval stratified by sex for each metabolite module, and separated by brain PLS-DA components. **(B)** Network of the heterogeneous modules between sexes, yellow, blue, and brown, indicating the correlation between the several metabolites. **(C)** Effect sizes in males and females across all brain PLS-DA components. Different types of metabolites are color coded.

Three metabolite modules in the p180 platform, the blue, brown, and yellow modules, were heterogeneous between sexes in brain component 4 (Figure 3 and Supplementary Table S1). Males showed a nominally significant positive association in the blue (*β* = 0.103, *P* = 0.009), brown (*β* = 0.084, *P* = 0.032), and yellow modules (*β* = 0.082, *P* = 0.038) indicating that lower levels of the blue, brown, and yellow module metabolites were associated with AD and MCI progression in brain component 4. The blue module contained 17 metabolites, including various alkylacyl (ae) and diacyl (aa) PCs and a single lysoPC (lysoPC a C24:0). The module membership (MM) values in the blue module were larger than 0.8 for all included metabolites, except for PC aa C38:5 and PC aa C40:5. Five metabolites, PC aa C40:3, PC ae C42:2, PC ae C44:3, PC ae C42:1, and PC ae C42:3, had MM values greater than 0.9. The yellow module contained eight metabolites, including various alkylacyl and diacyl PCs. The MM values for the yellow module were greater than 0.8 except for the only two diacyl PCs, PC aa C40:4 and PC aa C38:4. The most relevant metabolites in the yellow modules, with MM values greater than 0.9, were the alkylacyl PCs, PC ae C38:4, PC ae C38:5, PC ae C36:4, PC ae C40:4, and PC ae C40: 5. The brown module contained seventeen metabolites, mostly sphingomyelins and a few alkylacyl and diacyl PCs. All metabolites in the brown module had MM values greater than 0.8 except for the diacyl PC, PC aa C32:3, and the sphingomyelin SM C24:0. The most relevant metabolites, with MM values greater than 0.9, were the sphingomyelins SM (OH) C22:2, SM C16:0, and SM (OH) C16:1.

Forty-two metabolites were classified as heterogeneous between sexes when considering single metabolites, and 10 were sex-specific (Figure 3 and Supplementary Table S2). Of the heterogeneous metabolites, 13 showed nominally significant associations and greater effect sizes in females. These were mainly classified as acylcarnitines and were associated with brain components 3, 4, and 5. All these metabolites were included in the gray module except for one assigned to the yellow module. For example, increased levels of four acylcarnitines, C10:2, C7:DC, C14:1-OH, C9, were associated with AD progression in brain component 5 in females but not males. The remaining 29 metabolites out of the 42 classified as heterogenous were nominally significant and had greater effect sizes in males. These mainly were alkylacyl and diacyl PCs and were predominantly associated with brain component 4. However, several amino acids also showed a nominal significance in males. For example, lower aspartic acid, isoleucine, lysine, methionine, and valine levels were associated with AD and MCI progression in brain component 4 in males but not females. Similarly, lower citrulline, isoleucine, and tyrosine levels were associated with MCI progression in brain component 3 in males.

All 10 sex-specific metabolites were Bonferroni significant and had greater effect sizes in males, and were predominantly associated with brain components 2, 3, and 5. For example, lower levels of lysoPC a C20:4, PC ae C36:5, and arginine were associated with AD and MCI progression in brain component 2. On the other hand, lower levels of the biogenic amines creatinine and SDMA, and the acylcarnitine C10, were associated with AD progression in brain component 5.

#### 3.2.2. NMR Platform

**FIGURE 4 ∣:**
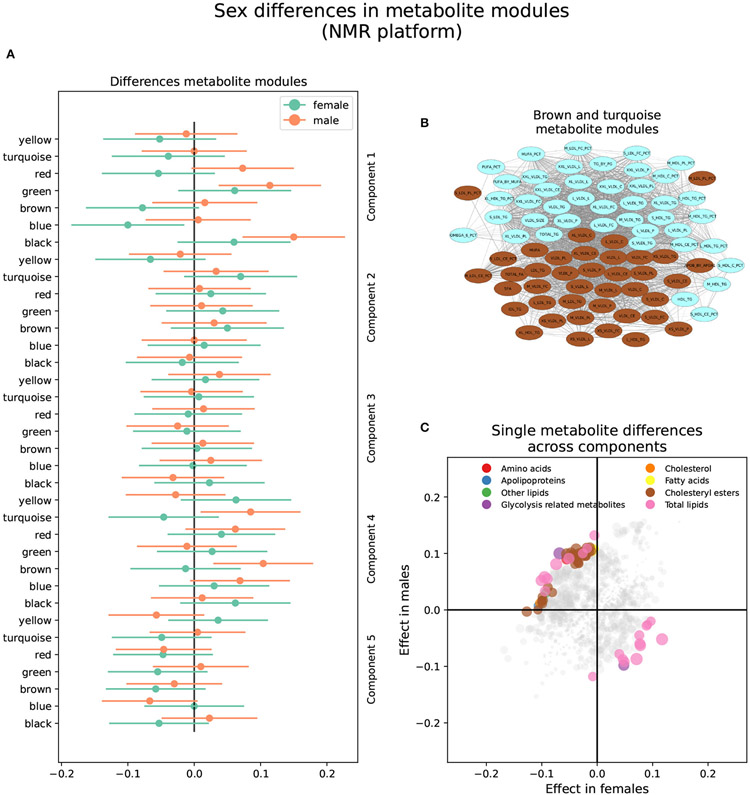
Sex differences in the NMR metabolomics platform. **(A)** Effect sizes and their 95% confidence interval stratified by sex for each metabolite module, and separated by brain PLS-DA components. **(B)** Network of the brown and turquoise modules indicating the correlation between all metabolites. **(C)** Effect sizes in males and females in each brain PLS-DA component. Different types of metabolites are color coded.

The brown and turquoise metabolite modules were heterogeneous between sexes in the NMR platform in brain component 2 (Figure 4 and Supplementary Table S3). The brown module showed a nominally significant positive association in males (*β_f_* = −0.013, *P_f_* = 0.76; *β_m_* = 0.104, *P_m_* = 0.0071), as well as the turquoise module (*β_f_* = −0.046, *P_f_* = 0.27; *β_m_* = 0.085, *P_m_* = 0.027; Figure 4A). In other words, increased brown and turquoise module metabolite levels were associated with AD and MCI progression in males but not females for brain component 2.

The brown module comprised 40 metabolites. It contained various lipoprotein subclasses, including triglycerides in IDL, large and medium LDL, large and very large HDL, and very small VLDL. It also contained cholesterol and cholesteryl esters in small, large, and very large VLDL, and free cholesterol, phospholipids, and total lipids in very small, small, and medium VLDL. Finally, among lipoprotein subclasses, it contained the concentration of very small, small, and medium VLDL particles. Different lipoprotein-to-lipid ratios were also included, such as cholesteryl esters and phospholipids to total lipid ratio in small and medium LDL. Only six metabolites in the brown module had MM values lower than 0.7, including all lipoprotein-to-lipid ratios, glycoprotein acetyls, and triglycerides in large HDL. On the other hand, the top five most relevant metabolites, with MM values higher than 0.97, were the concentration of VLDL particles, free cholesterol, cholesterol, phospholipids, and total lipids in VLDL, and total lipids in small VLDL.

The turquoise module was composed of 42 metabolites. Several lipoprotein subclasses were represented, specifically triglycerides in most subclasses of VLDL, small LDL, and small and medium HDL. Furthermore, it also contained free cholesterol, phospholipids, and total lipids in large, very large, chylomicrons and extremely large VLDL. Other lipoprotein subclasses, such as cholesterol and cholesteryl esters in chylomicrons and extremely large VLDL, and the concentration of large, very large, chylomicrons and extremely large VLDL particles were also included. Other metabolites included in the turquoise module were total triglycerides and triglycerides in HDL and VLDL, and various fatty acid ratios. Finally, several lipoprotein-to-lipid ratios were among the metabolites included in the turquoise module, specifically triglycerides to total lipids ratio in small, medium, large, and very large HDL, phospholipids to total lipids ratio in medium HDL, free cholesterol to total lipids ratio in small and medium LDL, and cholesterol and cholesteryl esters to total lipids ratio in small and medium HDL.

Only two metabolites had MM values less than 0.7, including the ratio of cholesterol and cholesteryl esters to total lipids in small HDL. On the other hand, the turquoise module’s most relevant metabolites, with MM values higher than 0.95, included the concentration of very large VLDL particles, total lipids, triglycerides, and phospholipids in very large VLDL, total triglycerides, and triglycerides in VLDL.

Seventy-three (73) single metabolites were heterogeneous, and one was classified as sex-specific in the NMR platform (Figure 4 and Supplementary Table S4). Of the heterogeneous metabolites, 27 were nominally significant and had greater effect sizes in females. These were primarily associated with brain components 1 and 5. Specifically, increased levels of triglycerides in HDL and IDL, glycerol, and triglycerides to total lipids ratio in small and medium LDL, as well as decreased levels of cholesterol, cholesteryl esters, and free cholesterol to total lipids ratio in LDL, were associated with AD progression in females in brain component 1. On the other hand, decreased levels of apolipoprotein B, remnant cholesterol, total cholesterol minus HDL-C, cholesterol, and cholesteryl esters in medium VLDL, among others, were associated with AD progression in brain component 5.

The remaining 46 heterogeneous metabolites were nominally significant and had greater effect sizes in males. Most were associated with brain component 2 and were included in the brown and turquoise modules. Besides those mentioned above, five metabolites were associated with brain component 1. Specifically, increased levels of free cholesterol to total lipids ratio in very small VLDL, phospholipids to total lipids ratio in small HDL, and triglycerides to total lipids ratio in large LDL, as well as decreased levels of triglycerides to total lipids ratio in very large VLDL, cholesterol to total lipids ratio in IDL, cholesterol to total lipids ratio in large LDL were associated with AD progression in males in brain component 1. Finally, four metabolites were associated with brain component 5. Specifically, increased levels of sphingomyelins, total phospholipids in lipoprotein particles, and free cholesterol to total lipids ratio in large and small HDL were associated with AD progression in males in brain component 5.

## 4. DISCUSSION

This study has identified sex differences in the association between metabolites and AD brain imaging phenotypes. Specifically, we have shown that diverse phosphatidylcholines (PC), sphingomyelins (SM), acylcarnitines, amino acids, and different lipids in very low-density lipoproteins (VLDL) and low-density lipoproteins (LDL) have different associations between men and women with AD progression. Our methodology highlights the beneficial use of diverse multivariate techniques to take advantage of the highly correlated structure of biological systems.

### 4.1. Brain Volume Changes in AD

The sex differences identified in this study were common across brain components. Brain component 1 contains the most common pattern of volumetric changes in AD , which includes atrophy of the hippocampus, entorhinal cortex, fusiform gyrus, middle temporal gyrus, and the whole brain, and an enlargement of the ventricles (28). Brain component 1 also explains most of the variance in brain volume, 64%, while the remaining brain components explain a similarly low percentage of the variance in brain volume and together account for only 30% of the variance. Although these lower components can separate diagnosis groups, the low percentage of variance explained can be interpreted as rare cases of brain volume changes due to AD or MCI. For example, brain component 2 only explains 10% of the variance and is characterized by relative atrophy of the hippocampus, entorhinal cortex, and ventricles and relative enlargement of the whole brain. However, because the brain segments are corrected by intracranial volume (ICV), caution needs to be taken when interpreting the contribution of the different segments. In this case, whole-brain enlargement can be better interpreted as a relatively greater proportion of the whole brain to ICV associated with AD progression. Furthermore, although the progression of AD and MCI from CN is at the core of the construction of the PLS-DA components, which is evident given the separation of diagnosis groups in brain component 1, the phenotypes addressed in this study are changes in brain volume and not diagnosis groups. In other words, changes in brain volume associated with AD or MCI progression might also be occurring in individuals without symptoms. The results from the PLS-DA emphasize the fact that different patterns of brain atrophy can emerge with cognitive decline and highlight the heterogeneity of AD (9). Furthermore, our study emphasizes the notion that unique patterns of cognitive decline are also relevant to sex differences. Most importantly, it highlights that depending on how AD progression is defined (i.e., what particular biomarkers, surveys, or imaging information are used), different patterns of sex differences can emerge.

### 4.2. Lipid Sex Differences in AD

One of the major categories in which we observed sex differences of metabolites with brain components was with various lipid categories. Triglycerides, PCs, SMs, acylcarnitines, and cholesterol showed differences in various brain components between sexes. Both PCs and SMs are central components of cellular membranes and neuronal membranes. PCs are a type of glycerophospholipid characterized by having a choline group in the *sn*-3 position (29). PCs can contain an ester-linked acyl chain in the *sn*-2 position, and can contain acyl-, ether-, or vinyl-ether bonds in the *sn*-1 position, and are classified into diacyl, alkylacyl or alkenylacyl PCs, respectively (30). On the other hand, SMs are one of the most common types of sphingolipids and are found in plasma, plasma lipoproteins, and cellular membranes (31).

Altered levels of PCs and SMs have been associated with AD development (32). However, several inconsistencies have been found (33). For example, levels of SM in the brain have been found to be greater (34, 35) and lower (36) in AD compared to normal controls. Similarly, SM in blood samples has been found to be lower (33, 37) and greater (35) when comparing AD or memory-impaired participants to controls. Similar inconsistent results have also been found in PC. Plasma PC have been found to be lower (38-42) and higher (6, 37) in AD or MCI compared to controls. Due to these discrepancies, it has been hypothesized that PC and SM have shifting roles during different stages of AD progression, with higher levels in pre-clinical stages and lower ones post-impairment (6, 43, 44). Although the specific mechanisms linking blood PC and SM to AD progression are not well understood, several hypotheses have been outlined. For example, PC and SM alteration have been implicated with the immune system (30, 45), providing another functional mechanism linking AD and inflammation (6, 46). Furthermore, PC and SM alterations have also been associated with other diseases known to be AD risk factors (47), such as diabetes mellitus (48), type 2 diabetes (49), insulin resistance (50), and cardiovascular risk factors like BMI and alcohol consumption (51).

This study found that lower plasma levels of diacyl and alkylacyl PC and SM are associated with AD and MCI progression in men but not women in brain component 4. However, we also found that increased levels of SM are associated with AD progression in brain component 5 in men only. Our findings, that sex differences exist in metabolite-AD associations but are specific to a brain volume pattern, might help reconcile previous inconsistent findings. For example, sample composition, specifically sex ratios, might affect the associations deemed significant and the ability to compare across studies. Furthermore, while a particular pattern of AD progression might be associated with increased lipid levels, a different pattern might show the opposite association. Our results also highlight the potential sex-specific nature of AD risk factors. For example, although diabetes has been shown to pose a greater risk in women compared to men in developing AD (52, 53), some studies have shown a stronger association between diabetes and MCI in men compared to women (54). Sex differences in lipid metabolism have been previously found. Particularly interesting is the find that alkylacyl PC has a stronger negative association with aging in men compared to women (55). Whether the concentration levels of PC and SM are a cause, effect, or how the different pathologies interact is beyond the scope of this study, but they highlight the need to take into account correlational structures that might be particular to each sex.

Increased levels of triglycerides have been associated with the development of AD and other forms of dementia, like vascular dementia (56-58). Due to the clear causal connection between hypertriglyceridemia and the development of atherosclerotic cardiovascular disease (ACVD) (59), there is an increased understanding of the role of triglycerides as shared risks factors with ACVD and dementia (57). Similarly, LDL, specifically LDL cholesterol content, has been implicated in cognitive impairment (60). Nevertheless, conflicting results have also been found for the role of triglycerides (60) and LDL (61) in AD. Our study indicates that lipid composition in LDL has differential associations between sexes with AD progression in brain component 1. Specifically, less cholesterol and more triglyceride content in LDL is associated with AD and MCI progression, but while women show a significant association only with small and medium LDL, men show a significant association only with large LDL. Our results highlight the complexity of the role of lipid contents of LDL on AD and the sex-specific role they might play.

The two metabolite modules classified as heterogeneous between sexes in the NMR platform can be interpreted together; while the brown module contains mostly small VLDL, the turquoise module contains large VLDL. However, while the top metabolites for the brown module were free cholesterol and cholesterol in VLDL, the principal metabolites in the turquoise module were triglycerides in VLDL. Therefore, different VLDL sizes and constituent lipids on VLDL impact sex differences. VLDLs are a class of triglyceride-rich lipoproteins whose function is to carry triglycerides synthesized in the liver to adipose tissue and muscle for energy production (62). Elevated VLDL, either due to an overproduction or failure in clearance, can lead to hypertriglyceridemia or an excess of triglycerides in blood (63). As mentioned before, increased triglycerides pose a risk of developing AD. Moreover, previous studies have found that lipid content, specifically cholesterol and triglycerides in VLDL, is associated with AD risk (64).

On the other hand, we also found that a reduction in cholesterol and increased triglycerides in VLDL is associated with AD progression in women in brain component 5. These seemingly contradictory results highlight the complexity of the role of lipids in AD progression. While the increase in lipids might be associated with a greater risk of AD development, the reduction of the same lipid might also be associated with an increased risk, either in a different pattern of AD progression or in a different sex.

### 4.3. Amino Acids Sex Differences in AD

Altered levels of amino acids are relevant in the process of aging, as well as aging-related diseases, including AD (65). Several amino acids have been shown to be altered in AD and MCI compared to controls, both in cerebrospinal fluid (CSF) and blood (66). For example, it has been found that polyamine, lysine, and tryptophan metabolism, and glycine and valine levels are altered in blood samples across AD, MCI, and CN groups (67, 68). Various studies have also explored the role of amino acid intake in the progression of AD; however, most research is still speculative due to the lack of specific underlying mechanisms (69). Our study found that decreased levels of arginine and serine are associated with AD and MCI progression in brain component 2 , and decreased levels of asparagine, methionine, and threonine are associated with MCI progression in brain component 3 in men but not women. These results underscore a potential heightened sensitivity to amino acid alteration in AD progression only in men. Authors have cautioned about the interpretation of amino acids due to their variation on nutritional status, specifically the difference in levels between fasting and non-fasting participants (67). In the case of our study, we retained only fasting individuals for analysis.

### 4.4. The Importance of Metabolomics Platforms

The two metabolomic platforms show different strengths and weaknesses when combined with WGCNA. In the p180 platform, even though we could detect three metabolite modules as heterogeneous between sexes, most were assigned to the gray module when evaluating single metabolite associations. On the other hand, we detected two metabolite modules as heterogeneous between sexes in the NMR platform, and when evaluating single metabolites, most (61%) sex different metabolites were already assigned to one of those modules. In other words, the use of WGCNA for this particular study was more successful in the NMR platform than in the p180 because it could assign relevant metabolites into modules. This difference is due to the correlation structure of the metabolites, which is very different between the two platforms. While the metabolites in the NMR platform showed a highly correlated structure, the metabolites in the p180, except for some highly correlated group of metabolites, generally showed a loose correlation structure. This difference might be due to the technical nature of the platforms; while NMR is an untargeted platform, p180 is a targeted one. The decision to include particular metabolites in the p180 platform needs to balance the coverage of distinct biological pathways with covering highly correlated metabolites in the same ones.

Even though we found sex differences using the approach of Arnold et al. (8), we were not able to find sex differences with the approach of Winkler et al. (21). The approach of Winkler et al. (21) is more stringent and has been tested through simulations and real data to evaluate valid type I error rates and power in the decision criteria. On the other hand, the approach of Arnold et al. (8) has not been evaluated under such strict testing.

### 4.5. Limitations

Our study has limitations. Notably, using dimension reduction techniques to define the phenotypes leads to a data-driven approach that can detect patterns in the data without using previous knowledge. On the other hand, the same strength leads to a much more challenging interpretation of the phenotype and limits the potential use in clinical settings. The lack of significant results using the Winkler et al. (21) approach indicates a potential lack of power to detect sex differences, mainly because the data needs to be stratified. Therefore, our results need to be taken with caution, and further replications will be needed to establish the significance of these conclusions.

However, our study presents several strengths. We evaluated the multidimensional aspect of AD progression, an underlying element in the differences in results across studies, and explicitly attempted to identify sex differences. Much research takes sex as a covariate to control for or as a research question secondary to primary analyses. Such a strategy might only be warranted when sample sizes do not allow for the stratification of the cohorts. However, when explicitly evaluating sex differences, common elements and particular results in each sex are easier to evaluate. Furthermore, in AD, as well as other diseases, it is clear that sex and gender play fundamental roles (70, 71).

## Supplementary Material

Data_Sheet_1_Sex Differences in the Metabolome of Alzheimer's Disease Progression.CSV

Data_Sheet_3_Sex Differences in the Metabolome of Alzheimer's Disease Progression.CSV

Data_Sheet_2_Sex Differences in the Metabolome of Alzheimer's Disease Progression.CSV

Image_1_Sex Differences in the Metabolome of Alzheimer's Disease Progression.TIF

Data_Sheet_4_Sex Differences in the Metabolome of Alzheimer's Disease Progression.CSV

## Figures and Tables

**TABLE 1 ∣:** Sample information.

		*APOEϵ*4		Age		Education	
Cohort	Sex	Category	Count	Mean	Std	Mean	Std
ADNI 1	Female	0	142	75.4	7.1	15.1	3.1
		1	104	74.0	6.0	14.5	3.0
		2	33	69.4	6.2	14.6	2.4
	Male	0	192	75.1	6.9	16.2	3.0
		1	151	75.5	6.6	15.8	3.1
		2	39	72.5	6.4	16.2	2.8
ADNI GO	Female	0	35	72.0	8.6	15.5	2.5
		1	15	68.0	8.4	14.5	3.0
		2	3	62.8	6.0	17.3	3.1
	Male	0	32	72.6	6.9	16.3	2.8
		1	24	71.3	7.2	16.2	2.4
		2	3	69.5	1.8	14.0	1.7
ADNI 2	Female	0	154	72.1	6.8	16.0	2.6
		1	111	70.2	6.2	15.6	2.6
		2	24	69.5	6.4	16.1	2.6
	Male	0	174	73.3	6.9	16.9	2.5
		1	97	73.2	7.0	16.9	2.4
		2	35	72.1	7.4	16.4	2.8

*Age and education average and standard deviation values stratified by ADNI cohort, sex, and APOEϵ*4 *condition. APOEϵ*4 *counts stratified by cohort and sex*.

## Data Availability

The data analyzed in this study is subject to the following licenses/restrictions: Data access requests should be submitted to the Alzheimer’s Disease Neuroimaging Initiative (ADNI). Requests to access these datasets should be directed to http://adni.loni.usc.edu/.

## References

[1] Alzheimer’s Association. 2018 Alzheimer’s disease facts and figures. Alzheimers Dement. (2018) 14:367–429. doi: 10.1016/j.jalz.2018.02.001

[2] Mazure CM, Swendsen J. Sex differences in Alzheimer’s disease and other dementias. Lancet Neurol. (2016) 15:451–2. doi: 10.1016/S1474-4422(16)00067-3 26987699 PMC4864429

[3] Altmann A, Tian L, Henderson VW, Greicius MD. Sex modifies the APOE-related risk of developing alzheimer disease. Ann Neurol. (2014) 75:563–73. doi: 10.1002/ana.24135 24623176 PMC4117990

[4] Ardekani BA, Convit A, Bachman AH. Analysis of the MIRIAD data shows sex differences in hippocampal atrophy progression. J Alzheimers Dis. (2016) 50:847–57. doi: 10.3233/JAD-150780 26836168

[5] Wilkins JM, Trushina E. Application of metabolomics in Alzheimer’s disease. Front Neurol. (2018) 8:719. doi: 10.3389/fneur.2017.00719 29375465 PMC5770363

[6] Toledo JB, Arnold M, Kastenmüller G, Chang R, Baillie RA, Han X, Metabolic network failures in Alzheimer’s disease: a biochemical road map. Alzheimers Dement. (2017) 13:965–984. doi: 10.1016/j.jalz.2017.01.020 28341160 PMC5866045

[7] Graham SF, Chevallier OP, Elliott CT, Hölscher C, Johnston J, McGuinness B, Untargeted metabolomic analysis of human plasma indicates differentially affected polyamine and L-arginine metabolism in mild cognitive impairment subjects converting to Alzheimer’s disease. PLoS ONE. (2015) 10:e0119452. doi: 10.1371/journal.pone.0119452 25803028 PMC4372431

[8] Arnold M, Nho K, Kueider-Paisley A, Massaro T, Huynh K, Brauner B, Sex and APOE *E*4 genotype modify the Alzheimer’s disease serum metabolome. Nat Commun. (2020) 11:1148. doi: 10.1038/s41467-020-14959-w 32123170 PMC7052223

[9] Poulakis K, Pereira JB, Mecocci P, Vellas B, Tsolaki M, Kłoszewska I, Heterogeneous patterns of brain atrophy in Alzheimer’s disease. Neurobiol Aging. (2018) 65:98–108. doi: 10.1016/j.neurobiolaging.2018.01.009 29455029

[10] St John-Williams L, Blach C, Toledo JB, Rotroff DM, Kim S, Klavins K, Targeted metabolomics and medication classification data from participants in the ADNI1 cohort. Scientific Data. (2017) 4:170140. doi: 10.1038/sdata.2017.140 29039849 PMC5644370

[11] Würtz P, Kangas AJ, Soininen P, Lawlor DA, Davey Smith G, Ala-Korpela M. Quantitative serum nuclear magnetic resonance metabolomics in large-scale epidemiology: a primer on -omic technologies. Am J Epidemiol. (2017) 186:1084–96. doi: 10.1093/aje/kwx016 29106475 PMC5860146

[12] Evans MC, Barnes J, Nielsen C, Kim LG, Clegg SL, Blair M, Volume changes in alzheimer’s disease and mild cognitive impairment: cognitive associations. Eur Radiol. (2010) 20:674–82. doi: 10.1007/s00330-009-1581-5 19760240

[13] Reuter M, Schmansky NJ, Rosas HD, Fischl B. Within-subject template estimation for unbiased longitudinal image analysis. Neuroimage. (2012) 61:1402–18. doi: 10.1016/j.neuroimage.2012.02.084 22430496 PMC3389460

[14] Lee LC, Liong CY, Jemain AA. Partial least squares-discriminant analysis (PLS-DA) for classification of high-dimensional (HD) data: a review of contemporary practice strategies and knowledge gaps. Analyst. (2018) 143:3526–39. doi: 10.1039/C8AN00599K 29947623

[15] Abdi H Partial Least Squares Regression and Projection on Latent Structure Regression (PLS Regression). Wiley Interdisc Rev. (2010) 2:97–106. doi: 10.1002/wics.51

[16] DiLeo MV, Strahan GD, den Bakker M, Hoekenga OA. Weighted correlation network analysis (WGCNA) applied to the tomato fruit metabolome. PLoS ONE. (2011) 6:e26683. doi: 10.1371/journal.pone.0026683 22039529 PMC3198806

[17] Pei G, Chen L, Zhang W. WGCNA application to proteomic and metabolomic data analysis. In: Shukla AK, editor. Methods Enzymol. Vol. 585 of Proteomics in Biology, Part A Austin, TX: Academic Press (2017). p. 135–58. 10.1016/bs.mie.2016.09.016 28109426

[18] Su Y, Wang J, Shi M, Niu X, Yu X, Gao L, Metabolomic and network analysis of astaxanthin-producing haematococcus pluvialis under various stress conditions. Bioresour Technol. (2014) 170:522–9. doi: 10.1016/j.biortech.2014.08.018 25164345

[19] Langfelder P, Zhang B, Horvath S. Defining clusters from a hierarchical cluster tree: the dynamic tree cut package for R. Bioinformatics. (2008) 24:719–20. doi: 10.1093/bioinformatics/btm563 18024473

[20] Langfelder P, Horvath S. WGCNA: an r package for weighted correlation network analysis. BMC Bioinformatics. (2008) 9:559. doi: 10.1186/1471-2105-9-559 19114008 PMC2631488

[21] Winkler TW, Justice AE, Cupples LA, Kronenberg F, Kutalik Z, Heid IM, Approaches to detect genetic effects that differ between two strata in genome-wide meta-analyses: recommendations based on a systematic evaluation. PLoS ONE. (2017) 12:e0181038. doi: 10.1371/journal.pone.0181038 28749953 PMC5531538

[22] Li J, Ji L. Adjusting multiple testing in multilocus analyses using the eigenvalues of a correlation matrix. Heredity. (2005) 95:221–7. doi: 10.1038/sj.hdy.6800717 16077740

[23] R Core Team. R: A Language and Environment for Statistical Computing. Vienna: R Foundation for Statistical Computing (2021).

[24] Lucas AM, Palmiero NE, McGuigan J, Passero K, Zhou J, Orie D, CLARITE facilitates the quality control and analysis process for ewas of metabolic-related traits. Front Genet. (2019) 10:1240. doi: 10.3389/fgene.2019.01240 31921293 PMC6930237

[25] Hunter JD. Matplotlib: A 2D Graphics environment. Comput Sci Eng. (2007) 9:90–5. doi: 10.1109/MCSE.2007.55

[26] Oliphant TE. Guide to NumPy: 2nd ed. Austin, TX: CreateSpace Independent Publishing Platform (2015).

[27] McKinney W Data structures for statistical computing in python. In: Proceedings of the 9th Python in Science Conference (Cambridge, MA). (2010). p. 51–6.

[28] Pini L, Pievani M, Bocchetta M, Altomare D, Bosco P, Cavedo E, Brain atrophy in alzheimer’s disease and aging. Ageing Res Rev. (2016) 30:25–48. doi: 10.1016/j.arr.2016.01.002 26827786

[29] Hishikawa D, Hashidate T, Shimizu T, Shindou H. Diversity and function of membrane glycerophospholipids generated by the remodeling pathway in mammalian cells. J Lipid Res. (2014) 55:799–807. doi: 10.1194/jlr.R046094 24646950 PMC3995458

[30] Dean JM, Lodhi IJ. Structural and functional roles of ether lipids. Protein Cell. (2018) 9:196–206. doi: 10.1007/s13238-017-0423-5 28523433 PMC5818364

[31] Kikas P, Chalikias G, Tziakas D. Cardiovascular implications of sphingomyelin presence in biological membranes. Eur Cardiol Rev. (2018) 13:42–45. doi: 10.15420/ecr.2017:20:3 PMC6159463 30310470

[32] Zhu TB, Zhang Z, Luo P, Wang SS, Peng Y, Chu SF, Lipid metabolism in Alzheimer’s disease. Brain Res Bull. (2019) 144:68–74. doi: 10.1016/j.brainresbull.2018.11.012 30472149

[33] Mielke MM, Lyketsos CG. Alterations of the sphingolipid pathway in Alzheimer’s disease: new biomarkers and treatment targets? Neuromolecular Med. (2010) 12:331–40. doi: 10.1007/s12017-010-8121-y 20571935 PMC3129545

[34] Bandaru VVR, Troncoso J, Wheeler D, Pletnikova O, Wang J, Conant K, ApoE4 disrupts sterol and sphingolipid metabolism in alzheimer’s but not normal brain. Neurobiol Aging. (2009) 30:591–9. doi: 10.1016/j.neurobiolaging.2007.07.024 17888544 PMC2758772

[35] Varma VR, Oommen AM, Varma S, Casanova R, An Y, Andrews RM, Brain and blood metabolite signatures of pathology and progression in Alzheimer disease: a targeted metabolomics study. PLoS Med. (2018) 15:e1002482. doi: 10.1371/journal.pmed.1002482 29370177 PMC5784884

[36] He X, Huang Y, Li B, Gong CX, Schuchman EH. Deregulation of sphingolipid metabolism in Alzheimer’s disease. Neurobiol Aging. (2010) 31:398–408. doi: 10.1016/j.neurobiolaging.2008.05.010 18547682 PMC2829762

[37] Liu Y, Thalamuthu A, Mather KA, Crawford J, Ulanova M, Wong MWK, Plasma lipidome is dysregulated in Alzheimer’s disease and is associated with disease risk genes. Transl Psychiatry. (2021) 11:1–18. doi: 10.1038/s41398-021-01362-2 34092785 PMC8180517

[38] Goodenowe DB, Cook LL, Liu J, Lu Y, Jayasinghe DA, Ahiahonu PWK, Peripheral ethanolamine plasmalogen deficiency: a logical causative factor in Alzheimer’s disease and dementia. J Lipid Res. (2007) 48:2485–98. doi: 10.1194/jlr.P700023-JLR200 17664527

[39] Han X Lipid alterations in the earliest clinically recognizable stage of Alzheimer’s disease: implication of the role of lipids in the pathogenesis of Alzheimer’s disease. Curr Alzheimer Res. (2005) 2:65–77. doi: 10.2174/1567205052772786 15977990

[40] Simpson BN, Kim M, Chuang YF, Beason-Held L, Kitner-Triolo M, Kraut M, Blood metabolite markers of cognitive performance and brain function in aging. J Cereb Blood Flow Metab. (2016) 36:1212–23. doi: 10.1177/0271678X15611678 26661209 PMC4929698

[41] Mapstone M, Cheema AK, Fiandaca MS, Zhong X, Mhyre TR, MacArthur LH, Plasma phospholipids identify antecedent memory impairment in older adults. Nat Med. (2014) 20:415–8. doi: 10.1038/nm.3466 24608097 PMC5360460

[42] Whiley L, Sen A, Heaton J, Proitsi P, García-Gómez D, Leung R, Evidence of altered phosphatidylcholine metabolism in Alzheimer’s disease. Neurobiol Aging. (2014) 35:271–8. doi: 10.1016/j.neurobiolaging.2013.08.001 24041970 PMC5866043

[43] Jiang Y, Zhu Z, Shi J, An Y, Zhang K, Wang Y, Metabolomics in the development and progression of dementia: a systematic review. Front Neurosci. (2019) 13:343. doi: 10.3389/fnins.2019.00343 31031585 PMC6474157

[44] Mielke MM, Bandaru VVR, Haughey NJ, Rabins PV, Lyketsos CG, Carlson MC. Serum sphingomyelins and ceramides are early predictors of memory impairment. Neurobiol Aging. (2010) 31:17–24. doi: 10.1016/j.neurobiolaging.2008.03.011 18455839 PMC2783210

[45] Crivelli SM, Giovagnoni C, Visseren L, Scheithauer AL, de Wit N, den Hoedt S, Sphingolipids in Alzheimer’s disease, how can we target them? Adv Drug Deliv Rev. (2020) 159:214–31. doi: 10.1016/j.addr.2019.12.003 31911096

[46] Heppner FL, Ransohoff RM, Becher B. Immune attack: the role of inflammation in Alzheimer disease. Nat Rev Neurosci. (2015) 16:358–72. doi: 10.1038/nrn3880 25991443

[47] Kling MA, Trojanowski JQ, Wolk DA, Lee VMY, Arnold SE. Vascular disease and dementias: paradigm shifts to drive research in new directions. Alzheimers Dement. (2013) 9:76–92. doi: 10.1016/j.jalz.2012.02.007 23183137 PMC3640817

[48] Zhang W, Sun G, Likhodii S, Aref-Eshghi E, Harper PE, Randell E, Metabolomic analysis of human synovial fluid and plasma reveals that phosphatidylcholine metabolism is associated with both osteoarthritis and diabetes mellitus. Metabolomics. (2016) 12:24. doi: 10.1007/s11306-015-0937-x 26708258

[49] Fikri AM, Smyth R, Kumar V, Al-Abadla Z, Abusnana S, Munday MR. Pre-diagnostic biomarkers of Type 2 diabetes identified in the UAE’s obese national population using targeted metabolomics. Sci Rep. (2020) 10:17616. doi: 10.1038/s41598-020-73384-7 33077739 PMC7572402

[50] Li Z, Zhang H, Liu J, Liang CP, Li Y, Li Y, Reducing plasma membrane sphingomyelin increases insulin sensitivity. Mol Cell Biol. (2011) 31:4205–18. doi: 10.1128/MCB.05893-11 21844222 PMC3187286

[51] Lacruz ME, Kluttig A, Tiller D, Medenwald D, Giegling I, Rujescu D, Cardiovascular risk factors associated with blood metabolite concentrations and their alterations during a 4-year period in a population-based cohort. Circ Cardiovasc Genet. (2016) 9:487–94. doi: 10.1161/CIRCGENETICS.116.001444 27784734

[52] Toro CA, Zhang L, Cao J, Cai D. Sex differences in Alzheimer’s disease: understanding the molecular impact. Brain Res. (2019) 1719:194–207. doi: 10.1016/j.brainres.2019.05.031 31129153 PMC6750802

[53] Gannon OJ, Robison LS, Custozzo AJ, Zuloaga KL. Sex differences in risk factors for vascular contributions to cognitive impairment & dementia. NeurochemInt. (2019) 127:38–55. doi: 10.1016/j.neuint.2018.11.014 30471324

[54] Roberts RO, Knopman DS, Geda YE, Cha RH, Pankratz VS, Baertlein L, Association of diabetes with amnestic and nonamnestic mild cognitive impairment. Alzheimers Dement. (2014) 10:18–26. doi: 10.1016/j.jalz.2013.01.001 23562428 PMC3830601

[55] Beyene HB, Olshansky G, Smith AAT, Giles C, Huynh K, Cinel M, High-Coverage plasma lipidomics reveals novel sex-specific lipidomic fingerprints of age and bmi: evidence from two large population cohort studies. PLoS Biol. (2020) 18:e3000870. doi: 10.1371/journal.pbio.3000870 32986697 PMC7544135

[56] Lee JY, Han K, Han E, Kim G, Cho H, Kim KJ, Risk of incident dementia according to metabolic health and obesity status in late life: a population-based cohort study. J Clin Endocrinol Metab. (2019) 104:2942–52. doi: 10.1210/jc.2018-01491 30802284

[57] Nordestgaard LT, Christoffersen M, Afzal S, Nordestgaard BG, Tybjærg-Hansen A, Frikke-Schmidt R. Triglycerides as a shared risk factor between dementia and atherosclerotic cardiovascular disease: a study of 125 727 individuals. Clin Chem. (2021) 67:245–55. doi: 10.1093/clinchem/hvaa269 33418579

[58] Raffaitin C, Gin H, Empana JP, Helmer C, Berr C, Tzourio C, Metabolic syndrome and risk for incident Alzheimer’s disease or vascular dementia: the three-city study. Diabetes Care. (2009) 32:169–74. doi: 10.2337/dc08-0272 18945929 PMC2606808

[59] Nordestgaard BG. Triglyceride-rich lipoproteins and atherosclerotic cardiovascular disease. Circ Res. (2016) 118:547–63. doi: 10.1161/CIRCRESAHA.115.306249 26892957

[60] Liu Y, Zhong X, Shen J, Jiao L, Tong J, Zhao W, Elevated serum TC and LDL-C levels in alzheimer’s disease and mild cognitive impairment: a meta-analysis study. Brain Res. (2020) 1727:146554. doi: 10.1016/j.brainres.2019.146554 31765631

[61] Reitz C, Tang MX, Manly J, Schupf N, Mayeux R, Luchsinger JA. Plasma lipid levels in the elderly are not associated with the risk of mild cognitive impairment. Dement Geriatr Cogn Disord. (2008) 25:232–7. doi: 10.1159/000115847 18264008 PMC2725016

[62] Gibbons GF, Wiggins D, Brown AM, Hebbachi AM. Synthesis and function of hepatic very-low-density lipoprotein. Biochem Soc Trans. (2004) 32:59–64. doi: 10.1042/bst0320059 14748713

[63] Packard CJ, Boren J, Taskinen MR. Causes and consequences of hypertriglyceridemia. Front Endocrinol. (2020) 11:252. doi: 10.3389/fendo.2020.00252 PMC7239992 32477261

[64] Tynkkynen J, Chouraki V, van der Lee SJ, Hernesniemi J, Yang Q, Li S, Association of branched-chain amino acids and other circulating metabolites with risk of incident dementia and Alzheimer’s disease: a prospective study in eight cohorts. Alzheimers Dement. (2018) 14:723–33. doi: 10.1016/j.jalz.2018.01.003 29519576 PMC6082422

[65] Canfield CA, Bradshaw PC. amino acids in the regulation of aging and aging-related diseases. Transl Med Aging. (2019) 3:70–89. doi: 10.1016/j.tma.2019.09.001

[66] Griffin JWD, Bradshaw PC. Amino acid catabolism in Alzheimer’s disease brain: friend or foe? Oxid Med Cell Longev. (2017) 2017:e5472792. doi: 10.1155/2017/5472792 PMC5316456 28261376

[67] Klavins K, Koal T, Dallmann G, Marksteiner J, Kemmler G, Humpel C. The ratio of phosphatidylcholines to lysophosphatidylcholines in plasma differentiates healthy controls from patients with Alzheimer’s disease and mild cognitive impairment. Alzheimers Dement. (2015) 1:295–302. doi: 10.1016/j.dadm.2015.05.003 PMC4700585 26744734

[68] Trushina E, Dutta T, Persson XMT, Mielke MM, Petersen RC. Identification of altered metabolic pathways in plasma and CSF in mild cognitive impairment and Alzheimer’s disease using metabolomics. PLoS ONE. (2013) 8:e63644. doi: 10.1371/journal.pone.0063644 23700429 PMC3658985

[69] Ozawa H, Miyazawa T, Miyazawa T. Effects of dietary food components on cognitive functions in older adults. Nutrients. (2021) 13:2804. doi: 10.3390/nu13082804 34444965 PMC8398286

[70] Podcasy JL, Epperson CN. Considering sex and gender in Alzheimer disease and other dementias. Dialogues Clin Neurosci. (2016) 18:437–46. doi: 10.31887/DCNS.2016.18.4/cepperson 28179815 PMC5286729

[71] Ferretti MT, Iulita MF, Cavedo E, Chiesa PA, Schumacher Dimech A, Santuccione Chadha A, Sex differences in Alzheimer disease—the gateway to precision medicine. Nat Rev Neurol. (2018) 14:457–69. doi: 10.1038/s41582-018-0032-9 29985474

