# Supplementary figures and images for: Sex Differences in the Metabolome of Alzheimer's Disease Progression
Source: Front Radiol. 2022 Mar 14;2:782864. doi: 10.3389/fradi.2022.782864 (PMC9014653; doi:10.3389/fradi.2022.782864)

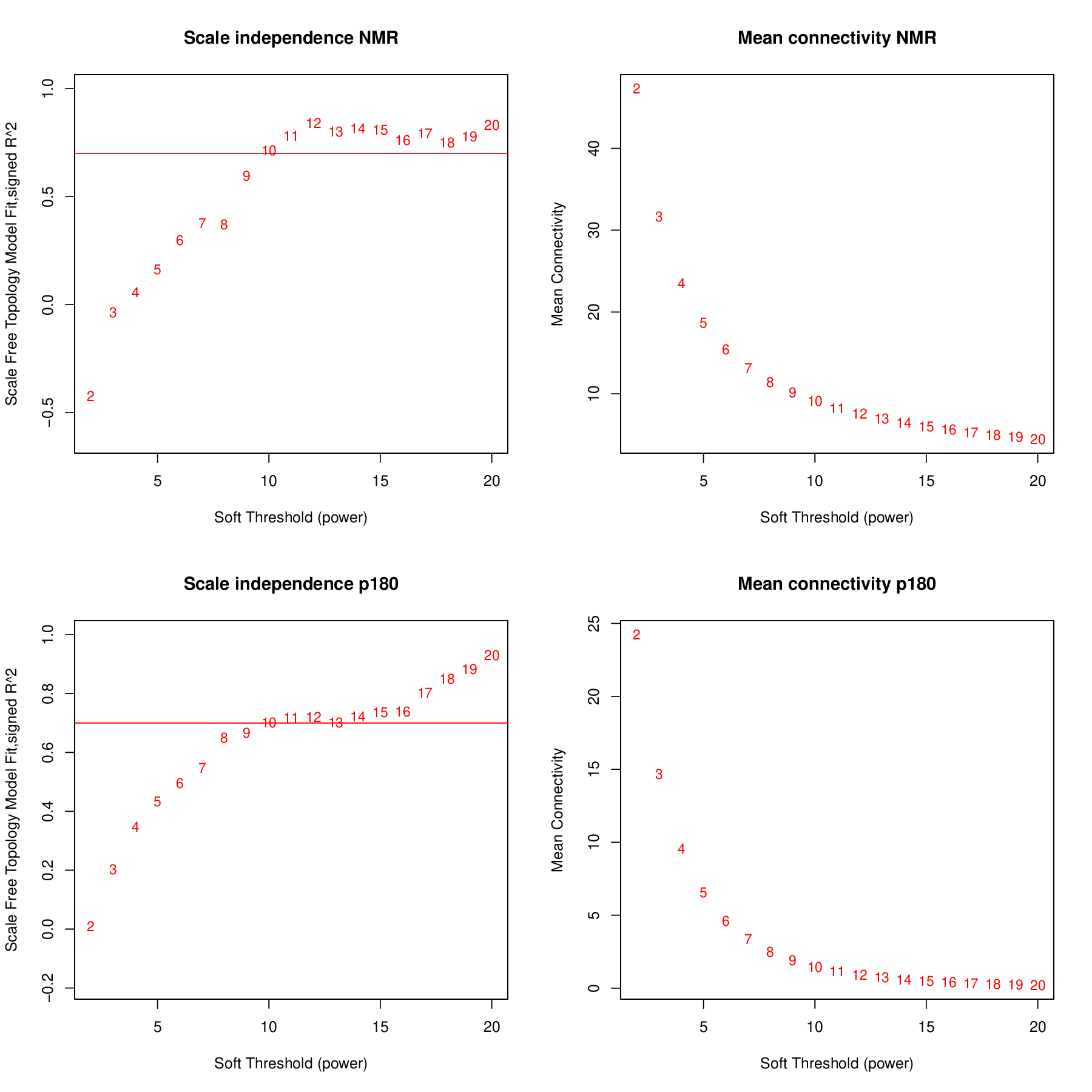

Supplement: Supplementary file 5 [file Image_1.TIF]
